# Molecular Investigations on Angiogenesis and Oxidative Stress in Roe Deer (*Capreolus capreolus*) Bucks' Testes Throughout the Reproductive Cycle

**DOI:** 10.1002/jez.70067

**Published:** 2026-01-27

**Authors:** Ilaria Troisio, Domenico Ventrella, Bálint Lóránt Hausz, Mattia Cesauri, Niccolò Ian Vannetti, Maria Laura Bacci, Alberto Elmi, Augusta Zannoni

**Affiliations:** ^1^ Department of Veterinary Medical Sciences University of Bologna Bologna Italy; ^2^ Health Science and Technologies Interdepartmental Center for Industrial Research (CIRI‐SDV) University of Bologna Bologna Italy; ^3^ Department of Veterinary Science University of Pisa Pisa Italy

**Keywords:** angiogenesis, NRP2, oxidative stress, Roe deer, SOD3, spermatogenesis suspension, testicular remodeling

## Abstract

Animals with seasonal reproductive cycles, as the Roe deer (Capreolus capreolus), have developed mechanisms to synchronize reproduction with the environmental cycle in order to optimize reproductive success through melatonin. Angiogenesis and oxidative stress are key processes in spermatogenesis, contributing to testicular remodeling and recovery after reproductive effort. This study carried out a gene expression analysis on 18 samples of mature male Roe deer testicles, collected during the local hunting season in pre‐rut (*N* = 9) and post‐rut (*N* = 9) periods. A quantitative real‐time PCR (qPCR) array targeting 84 genes involved in oxidative stress and 84 in angiogenesis were used, followed by validation through individual qPCR of selected genes and related protein quantification by ELISA assays. Post‐rut animals showed upregulation of several antioxidant genes: Peroxiredoxin‐4 (PRDX4), Scavenger receptors class A member 3 (SCARA3), Superoxide Dismutase 3 (SOD3). Instead, Leptin (LEP) and Thrombospondin Ⅱ (THBSⅡ), a known angiogenesis inhibitor, are downregulated. A novel insight is represented by the upregulation of Neuropilin (NRP2) in post‐rut period that, given to its posttranscriptional silencing too, needs better investigations. The pleiotropic nature of NRP2, including roles in neurodevelopment, immune modulation, and vascular remodeling, makes this gene an interesting candidate for further study, cause its function in reproductive tissues remains poorly understood.

## Summary

- Gene expression in testicular tissue varies between pre‐rut and post‐rut periods, reflecting physiological adaptations to seasonal reproduction.
- The study combined qPCR arrays and ELISA‐based protein validation to provide a robust analysis of oxidative stress and angiogenesis pathways in seasonal spermatogenesis.
- Post‐rut upregulation of NRP2, coupled with evidence of translational repression, suggests a complex role worthy of further investigation in reproductive biology.

## 1 Introduction

The synchronization between reproduction and the environmental cycle has always been a key evolutionary factor for reproductive success in many mammals living in non‐equatorial regions (Zerbe et al. 2012). As is well known, in many mammals, reproduction is regulated by the photoperiod (Chemineau et al. 2008), since the light stimulus triggers a hormonal response through melatonin action, responsible for pulsatile secretion of GnRH and gonadotropins (Dardente and Simonneaux 2022). Short‐day species (e.g., sheep and goats) reproduce in autumn, while long‐day species (e.g., horses) reproduce in spring. In these, we witness a reduction in gonadal activity and a return to anoestrus at the end of the breeding season (Chemineau et al. 2008).

For what specifically concerns the Roe deer (*Capreolus capreolus*), the reproductive season of sexually mature males, called bucks, can be easily categorized into three well‐distinct periods, during which substantial changes in size and consistency of the reproductive tract occur: pre‐rutting (from May to mid‐July), rutting period (from mid‐July to mid‐August), and post‐rutting (from mid‐August to September) (Klonisch et al. 2006). These seasonal variations can be perceived as adaptations to reduce the energy costs associated with reproduction; indeed, during the winter, bucks show a complete suspension of spermatogenesis. This natural pattern makes the Roe deer an interesting model for studying the seasonal regulation of spermatogenesis under the influence of photoperiod, an area of research already initiated by this research group (Elmi et al. 2021, 2020). Other works already highlighted changes particularly pronounced in size and consistency of reproductive traits during the year, particularly pronounced in testicle size and volume, as could be easily assessed also by an eye evaluation (Goeritz et al. 2003). Moreover, fluctuations in density and volume of the ejaculates with a peak during the rutting period are present (Goeritz et al. 2003). These evidences are also supported by the fluctuation of testosterone, essential for normal spermatogenesis and prevention of apoptotic cell death in androgen‐dependent tissues, which presents its highest level in serum and testis between late July and August (the maximum period of meiosis activity in Roe deer) with a sharply decrease and absence during the winter, when there is a complete spermatogenesis suspension (Roelants et al. 2002). Accordingly, it is also proven that LH and FSH gonadotropins, essentials for the activation of Leydig cells' differentiation and Sertoli cells' activation, respectively, show their maximum levels in blood in March with the purpose of maintaining testicular function and preparing the right environment for correct spermatozoa's maturation (Roelants et al. 2002).

It is well acknowledged how the process of angiogenesis, tightly regulated by Vascular Endothelial Growth Factor (VEGF), plays a fundamental role in spermatogenesis by balancing the growth and development of testicular tissue (Caires et al. 2009; Ebisch et al. 2008; Nalbandian et al. 2003). Another role is played by oxidative stress since DNA damage, apoptosis, and epigenetic alterations can be observed in the case of massive activation of reactive oxygen species (ROS) not balanced by antioxidant defense system. The disruption of this fragile balance may undermine the survival of germ cells, activating apoptotic pathways, thereby compromising the entire process of spermatogenesis (Ayad et al. 2022; Takeshima et al. 2020; Aitken and Clarkson 1987). Therefore, the aim of this work was to investigate the differential behavior of these critical pathways in testicular samples collected during the pre‐rut and post‐rut periods, both at gene expression and protein levels.

## 2 Materials and Methods

### 2.1 Animals

The samples included in this study were collected from 18 bucks (*N *= 18) during the 2018 hunting season in the Southwestern Bologna Apennines (Italy). Half of them were hunted during the pre‐rut period (June 1 to July 15; *n* = 9), while the other half during post‐rut period (August 15 to September 30; *n* = 9). To avoid any bias due to possible pathological conditions, animals found dead during the sampling activity were not included in this study. Immediately after their death, carcasses were conferred to local biometric center where scrota, including testicles, were collected from the personnel and, within 2 h, were transferred, at refrigerated temperature (5 ± 1°C), to the laboratories of Department of Veterinary Medical Science of the University of Bologna (Ozzano dell'Emilia, Italy) (Elmi et al. 2021, 2020). Ages were assessed upon teeth eruption and wear patterns evaluations as previously described (Elmi et al. 2020). Since all biological specimens were obtained from hunted animals (in accordance with local legislation), no ethical approval was required.

### 2.2 Testicular Tissue Sampling

The detailed methodology for collection and storage of the testicular parenchyma has been previously described (Elmi et al. 2020). Briefly, after the isolation from scrota, testes were weighed and cut in half to collect the middle section of parenchyma, minced, and split into aliquots. For gene expression analyses, aliquots were stored in RNA Stabilization Solution (RNAlater, Thermo Fisher Scientific, Waltham, MA, USA) for 24 h at +4°C and then moved at −80°C after solution removal (for RNA extraction). As for protein assay, aliquots were snap‐frozen in liquid nitrogen and immediately stored at −80°C.

### 2.3 Homogenization and Extraction of Total RNA and qPCRs Arrays on Pooled Samples

Tissues were firstly homogenized in TRIzolTM Reagent (30 mg/mL) (Molecular Research Center Inc., Cincinnati, OH, USA) and the resulting suspensions were used for total RNA extraction using NucleoSpin RNA (MACHEREY*‐*NAGEL GmbH & Co. KG, Düren, Germany), following the manufacturer's instructions as previously described (Elmi et al. 2020). The RNA quantity was assessed by spectrophotometry measuring the A260/A280 ratio using Nanospectrophotometer (Denovix Inc. Wilmington, DE, USA) followed by a quality evaluation assessment by gel electrophoresis on a 1% agarose gel. After quantification, two different pools (pre‐rut and post‐rut) were prepared to obtain a final concentration of 250 ng/µL for each of them. The reverse‐transcribed total RNA to cDNA was obtained using RT^2^ First Strand Kit (QIAGEN Hilden, Germany).

For each pool, Cow Angiogenesis RT^2^ Profiler PCR array (QIAGEN) and Cow Oxidative stress RT^2^ Profiler PCR array (QIAGEN) were performed according to the manufacturer's instructions, using 2X RT^2^ SYBR Green Mastermix (QIAGEN), and CFX 96 Touch (Bio‐Rad Laboratories, Hercules, CA, USA). Each array contained primers for 84 target genes and five housekeeping genes (HKG); seven wells included reverse‐transcription controls (RTC), positive PCR controls (PPC), and a genomic DNA contamination control. Gene expression was evaluated using the Δ*C*_t_ method (mean reference genes *C*_t_—interest gene *C*_t_), according to the RT2 Profiler PCR Array Handbook.

As a parameter of quantitative gene expression, the threshold cycle (*C*_t_) was used, which is inversely proportional to the amount of template present in the wells. Only genes with *C*_t_ values < 35 were reported and evaluated by using the ∆*C*_t_ (∆ threshold cycle) method, calculated as (∆*C*_t_ = *C*_t_ mean reference genes–*C*_t_ gene of interest). Gene expression was assessed using the fold‐change method ($2^{∆∆C_{t}}$), where ∆∆*C*_t_ = ∆*C*_t_ post‐rut−∆*C*_t_ pre‐rut, quantifying expression variations between the two groups (post vs. pre).

### 2.4 qPCR for the Quantification of Selected Genes on Single Animals

To validate the results obtained from the array performed on the pools, a single qPCR reaction (*N* = 18) was performed for the genes whose expression changes at least three times (up or down, post vs. pre) by using specific primers (QIAGEN). All samples were loaded in duplicate, and qPCRs were performed following the same procedure as before for each HKG and gene of interest. The results are also reported in this case using the fold‐change method ($2^{∆∆C}t$) normalized on the arithmetic mean of the three reference genes (ACTB, B2M, YWHAZ). Primer for reference genes were purchased from QIAGEN (RT2 qPCR Primer Assay for Beta‐Actin ‐ACTB; Beta‐2‐Microglobulin ‐B2M; ‐and tyrosine 3‐monooxygenase/tryptophan 5‐monooxygenase activation protein zeta ‐YWHAZ‐, Cat. No.; PPB00173A; PPS00031A, PPB01343A, respectively).

The genes of interest of individual analysis were 24‐Dehydrocholesterol reductase (DHCR24), Peroxiredoxin‐4 (PRDX4), Superoxide Dismutase 3 (SOD3), scavenger receptors class A member 3 (SCARA3) belonging to oxidative stress array, and Coagulation Factor II (F2), Leptin (LEP), Thrombospondin Ⅱ (THBSⅡ), Neuropilin 2 (NRP2) belonging to angiogenesis array. Primers for genes of interest were purchased from QIAGEN (Hilden, Germany). RT2 qPCR Primer Assay: DHCR24 (Cat. No. PPB09699A); PRDX24 (Cat. No. PPB00807A); SOD3 (Cat. No. PPB09315A); SCARA3 (Cat. No. PPB12336A); F2 (Cat. No. PPB00005A‐200); LEP (Cat. No. PPB00093A); THBSⅡ (Cat. No. PPB01550A); NRP2 (Cat. No. PPB12260A‐200).

In addition to Roe deer samples, RNA extraction and qPCR were also performed, when needed, on a bovine testis as a positive control. The specificity of the amplified PCR products was confirmed by agarose gel electrophoresis and melting curve analysis.

### 2.5 Enzyme‐Linked Immunosorbent Assay (ELISA)

According to the qPCR results, we performed the quantification of SOD 3, DHCR24, NRP2, and LEP proteins in testicular tissue using commercially available ELISA Kits (SOD3: MBS062357‐96; DHCR24: MBS9349018; NRP2: MBS2802336; LEP: MBS743525 from MyBiosource Inc., San Diego, USA) following the manufacturer's instructions. Tissues were homogenized in phosphate‐buffered saline (PBS) according to the dilution ratios suggested by the manufacturer: 1:10 for NRP2, DHCR24, and SOD3, 4:5 for leptin. Absorbance was measured at 450 nm using a Tecan Infinite F50 plate reader equipped with Magellan software (Tecan Group Ltd., Männedorf, Switzerland), and concentrations were calculated based on standard curve readings. All samples were run in duplicates.

### 2.6 Statistical Analysis

Descriptive statistics were calculated and reported as means. Data were assessed for normality of distribution using the Shapiro−Wilk test and further analyzed by means of a parametric or nonparametric test accordingly. Welch's *t*‐test was used to assess the statistical significance of gene expression differences between the pre‐rut and post‐rut animal groups. Data from ELISAs were analyzed using an unpaired *t*‐test to determine statistically significant differences between the two experimental groups. To evaluate relationships between gene expression and its corresponding protein content, a nonparametric Spearman's rank correlation test was performed. Statistical evaluations and graphic representations were obtained using GraphPad Prism v.8 (GraphPad Software Inc., San Diego, CA, USA). Significance was set for *p* < 0.05.

## 3 Results

### 3.1 Results of qPCR Arrays on Pooled Samples, Quality Control, and Normalization of Data

Following the qPCR protocol, to confirm that the amplification of the samples occurred specifically, the next step was the evaluation of melting curves, assessing the Tm of the amplified product and the *C*_t_ of each gene to evaluate the stability and specificity of the amplification. All the *C*_t_ recorded are reported in Supporting Information S1: Table S1 (oxidative stress) and Supporting Information S1: Table S2 (angiogenesis). Among the 84 genes analyzed, 54 genes related to the oxidative stress pathway and 56 genes related to the angiogenesis pathway were detectable, with *C*_t_ values below 35 in both groups. Following the kit's instructions, all genes with *C*_t_ values greater than 35 were excluded from further analysis, as this threshold is near the limit of detection. A few other genes were found to be non‐detectable (N/A) and were not included in the final total number of genes represented in heat maps.

∆CT values were used to compare reverse RTC and PPC for both pathways of this study. ∆CT values obtained ( < 5) suggest no impurities in samples that could have interfered with reverse transcription. CT^PPC^ values fall within the correct range of 20 ± 2, indicating that PCR conditions could be considered appropriate. The normalization of samples' CT values occurred against HKG.

**Figure 1:**
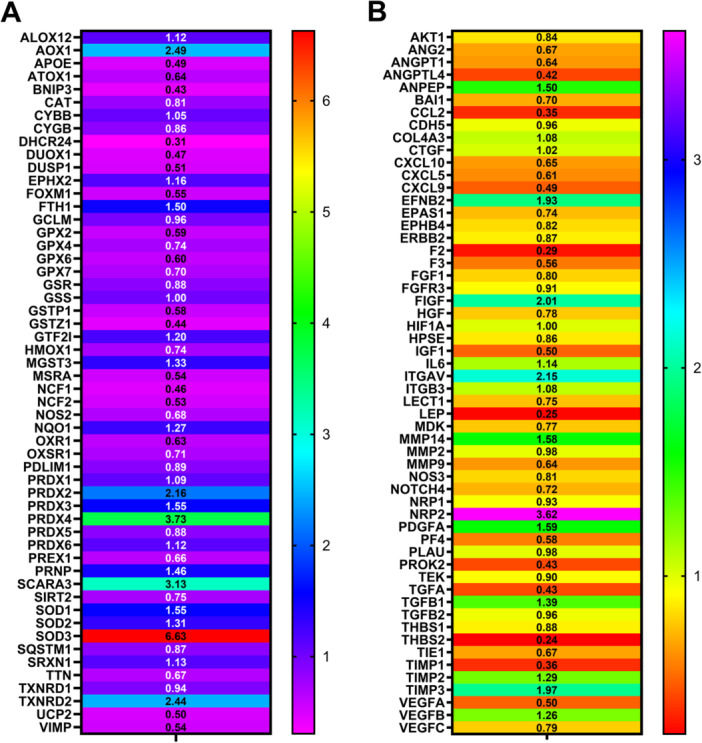
$2^{∆∆C}t$ post‐rut versus pre‐rut: ${2^{∆∆C}}_{t}$ oxidative stress assay (A); $2^{∆∆C}t$ angiogenesis assay (B).

∆CT values of pre‐rut group and post‐rut group (gene < 35 *C*_t_) of each pathway are reported in the Heat maps (Supporting Information S1: Figure 1).

To assess variations among groups, data is presented as $2^{∆∆C}T$, where ∆∆CT = ∆CT post‐rut−∆CT pre‐rut. The fold change > 1 or < 1 values indicate, respectively, an increase or decrease in gene expression in post‐rut in relation to the pre‐rut (control, value = 1) (Figure 1).

For both pathways, we select genes whose expression varied, in term of $2^{∆∆C}T$ value at least ±3 times. Figure 1A shows how the genes of the oxidative stress assay, whose expression varied by at least three times were 4: 24‐Dehydrocholesterol Reductase (DHCR24) downregulated by ~0.30 times and Peroxiredoxin 4 (PRDX4), Scavenger receptor class A member 3 (SCARA3), Superoxide dismutase (SOD3) upregulated, respectively, ~ 3.73; ~ 3.14; and ~ 6.63 times.

For the angiogenesis assay too, four genes varied their expression ±3 times. Three are downregulated: Coagulator factor Ⅱ (F2) ~0.29; Leptin (LEP) ~0.25; Thrombospondin 2 (THBSⅡ) ~0.24; while only one was upregulated: Neuropilin 2 (NRP2) ~3.62.

### 3.2 Results of qPCR for the Quantification of Selected Genes on Single Animals

**Figure 2:**
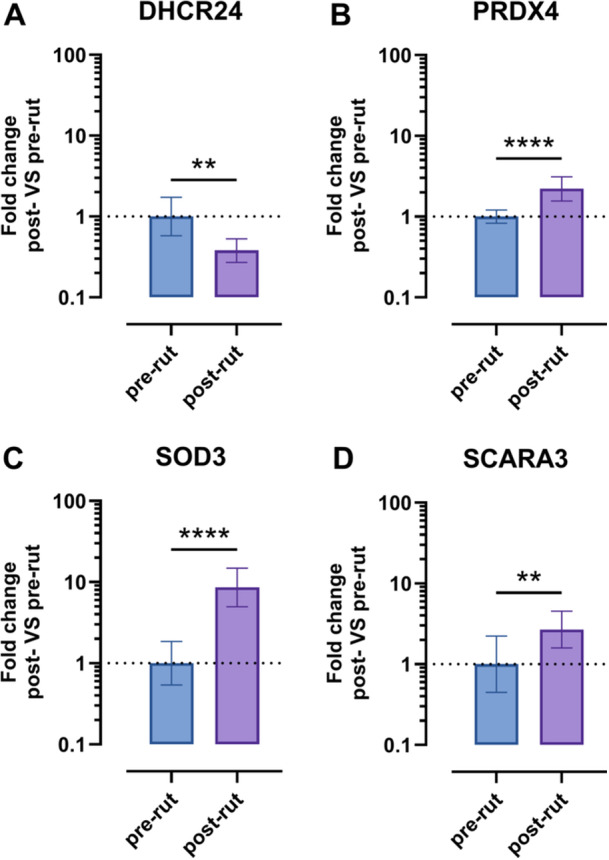
Oxidative stress panel. Data are reported on log10 scales. DHCR24 (A), PRDX4 (B), SOD3 (C), SCARA3 (D); **p < 0.01, ****p < 0.0001.

**Figure 3:**
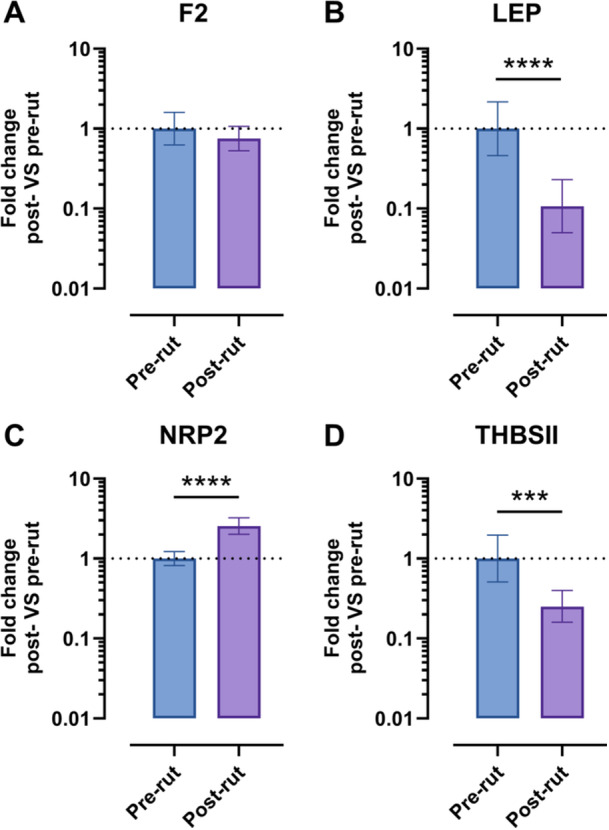
Angiogenesis panel. Data are reported on log10 scales. F2 (A), LEP (B), NRP2 (C), THBSII (D); ***p < 0.001, ****p < 0.0001.

The results of the single qPCRs conducted on each animal for the genes whose expression varied ±3 times are reported in the following figures, plated according to the specific pathway: Figure 2 (oxidative stress), Figure 3 (angiogenesis). Results are presented as fold of change post‐rut single animal versus pre‐rut single animal.

The results of the oxidative stress pathway (Figure 2) present statistically significant variations in gene expression among the two groups. Graphs show a decrease in the expression of DHCR24 in the post‐rut group (*p* = 0.0018) (Figure 2A). On the other hand, an upregulation of PRDX4 (*p* < 0.0001) (Figure 2B) is clear. At the same time, a sharp increase of about eight times can be noted for SOD3 (*p* < 0.0001) (Figure 2C), while SCARA3 is upregulated about three times (*p* = 0.0018) (Figure 2D).

Figure 3 reports the results of the second pathway analyzed: angiogenesis. A decrease in F2 expression in post‐rut group is clear, yet not statistically relevant (Figure 3A). Instead, the decrease in LEP after the rutting period (*p* < 0.0001), as well as THBSⅡ (*p* = 0.002) is statistically verified (Figure 3B,C). The only gene showing an increment in the post‐rut is NRP2, with an upregulation of 2.5 times (*p *< 0.0001) (Figure 3D).

### 3.3 Results of the ELISAs

**Figure 4:**
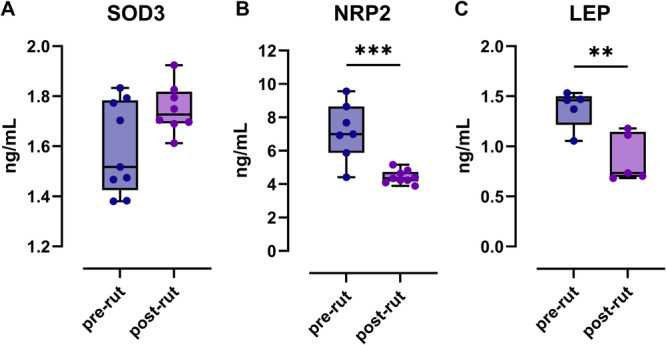
SOD3 (A), NRP2 (B), LEP (C); **p < 0.01, ***p < 0.001.

The results of protein quantification are shown in Figure 4, presented as box and whiskers plots. Despite following the manufacturer's instructions, all samples assayed for the quantification of DHCR24 were below the sensitivity of the test and were therefore considered unreliable.

Figure 4A shows an increase in SOD3 protein concentration in post‐rut animals without relevance from a statistical point of view. Instead, the decrease in NRP2 (Figure 4B) and leptin concentrations (Figure 4C) in the post‐rut animals is statistically relevant, with *p* of 0.0069 and 0.0004, respectively.

### 3.4 Correlation Analysis

**Figure 5:**
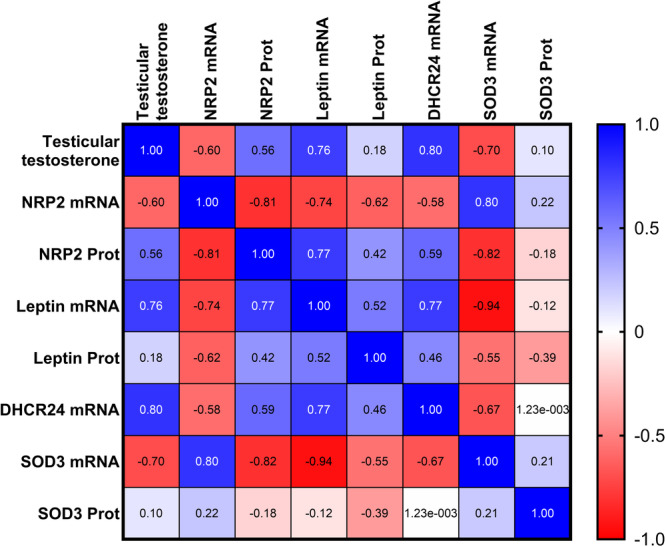
Spearman's rank correlation coefficients (*ρ*) table for the analyzed parameters, including previously published testicular testosterone (Elmi et al. 2020). Looking at the correlations between gene expression and protein level of the same analyte, NRP2 shows a significant negative correlation (*ρ* −0.81; *p* < 0.001), while the other analytes show positive relationships, yet not statistically relevant.

The results of the nonparametric Spearman's correlation analysis (expressed as *ρ*) are represented in Figure 5. The table also includes testicular testosterone levels, indicative of the activity of the testes at the moment of sampling, assessed for a previous study of the research team (Elmi et al. 2020).

NRP2 mRNA is positively correlated with SOD3 mRNA (*ρ* 0.80; *p* < 0.0001) and negatively with leptin mRNA (*ρ* −0.74; *p* < 0.001). The latter also shows a negative correlation with SOD3 mRNA (*ρ* −0.94; *p* < 0.0001), which is itself negatively correlated to DHCR24 mRNA (*ρ* −0.67; *p* < 0.01). Finally, testicular testosterone shows strong positive correlations with DHCR24 mRNA (*ρ* 0.80; *p* < 0.001) and leptin mRNA expression (*ρ* 0.76; *p* < 0.001), and negative ones with SOD3 mRNA (*ρ* –0.70; *p* < 0.01) and NRP2 mRNA (*ρ* −0.60; *p* < 0.01).

## 4 Discussion

The distinctive and temporally restricted regulation of spermatogenesis in Roe deer makes it a valuable model deserving deeper exploration, both for understanding species‐specific reproductive strategies and for comparative studies. Although literature is witnessing a growing interest in such a field (Kozioł et al. 2020; González‐Arto et al. 2016; Wagener et al. 2010; Klonisch et al. 2006; Roelants et al. 2002), many aspects still require clarification. Thus, samples of testicular parenchyma owned to 18 animals hunted in pre‐ and post‐rut period have been included in this study aimed at deepening the knowledge on this finely regulated mechanism using gene and protein expression methodologies.

Gene expression results from angiogenesis and oxidative stress arrays are shown as heat maps (Figure 1). First, mean ∆CT values of the pre‐ and post‐rut pools groups for genes with CT cycle < 35 (Supporting Information S1: Figure 1), then relative gene expression changes are reported using a $2^{∆∆C_{t}}$ method to present results in a clear and standardized way, allowing for comparison (Figure 1). This approach enabled selection of genes which expression varied at least ±3 times, later quantified singularly on a single animal (Figures 2 and 3).

As shown in Figure 2, 4, four genes (DHCR24, PRDX4, SOD3, and SCARA3) belonging to the oxidative stress pathway showed statistically significant variations in fold change expression in the post‐rut pool compared to the pre‐rut group, considered as the control reference. DCHR24, widely expressed in steroidogenic tissues such as testicular parenchyma (Peri 2016; Zerenturk et al. 2013), encodes a key enzyme involved in final step of cholesterol biosynthesis (London et al. 2015). Its gene expression downregulation in the post‐rut period in Roe deer reflects the seasonal decline of androgen levels. Moreover, since seasonal testicular involution is mainly due to reduced spermatogonia proliferation rather than widespread apoptosis (Elmi et al. 2021; Blottner et al. 2007), a reducing DHCR24 expression could reflect a controlled slowing down of metabolism, preserving testicular integrity for the next breeding season. Despite acting through and within diverse mechanisms, the remaining genes belonging to the oxidative stress panel (PRDX4, SOD3, and SCARA3) are involved in antioxidative processes and showed an increase in expression, especially with regard to SOD3 (about eightfold) in pre‐rout group. PRDX4 gene encodes for PRDX4 protein, a member of the peroxiredoxin family, that are redox‐active enzymes reducing peroxides, particularly hydrogen peroxide (H₂O₂). A testis‐specific PRDX4 isoform, PRDX4t, is expressed exclusively in sexually mature testes of many animals and is crucial for sperm nuclear protection during epididymal maturation, aiding in the oxidative folding of protamine (Fujii et al. 2025). SOD3, instead, encodes for glycoprotein SOD3 belonging to a group of metalloenzymes, known as SODs, which play a pivotal role in defending mechanism against ROS. Additionally, SOD3 is predominantly found in the extracellular matrix, potentially supporting areas not accessible to other SODs family members (Kalmari and Colagar 2024). Previous studies analyzing the mRNA expression of this gene in the Cervidae family highlighted significantly higher expression levels during the rutting period, when compared to the pre‐rut one in Roe deer epididymis (Koziorowska‐Gilun et al. 2015) and to the no‐rutting period in European Red deer (Neuman et al. 2025). However, in the latter, despite the mRNA abundance of SOD3 being consistently highest in the testis, no statistically significant seasonal differences were observed (Neuman et al. 2025), which contrasts with the clear seasonal variation indicated by our results. It is important to note that the European Roe deer does not exhibit a complete suspension of spermatogenesis throughout the year, which may limit its suitability as a model for studying seasonal gene expression changes in testis related to reproductive cycles. In the oxidative stress pathway (Figure 2), a marked post‐rut versus pre‐rut increment of SOD3 is shown (Figure 2C), likely reflecting a critical role in testes recovery, following the massive reproductive effort of the preceding rutting period, also supported by the same trend in PRDX4 (Figure 2B). Looking at the SOD protein levels measured by ELISA, the results mirror the mRNA expression, yet without statistical significance (Figure 4A). SCARA3 is a class A scavenger receptor and is considered a cellular stress response gene (Peng et al. 2020; Yap et al. 2015). To the best of our knowledge, there are no previous evidence for a specific role in the reproductive context in wild mammals; therefore, our findings may represent the first indications of SCARA3 in such a setting. The statistically relevant increment in term of gene expression shown in post‐rut group (Figure 2D) is consistent with what was already highlighted for previous genes included in the same panel involved in antioxidant process too.

The angiogenesis plate instead (Figure 3) shows four genes belonging to the same PCR array: F2, LEP, NRP2, and THBSⅡ. In this second panel, not all the genes show significant changes in terms of fold change expression post‐rut versus pre‐rut when analyzed individually on singular animals. For example, F2, encoding prothrombin which is a key component of the coagulation cascade (Chinnaraj et al. 2018), shows a decrease in gene expression in post‐rut pooled samples, yet not statistically significant at the individual level (Figure 3A). On the other hand, a strong statistically significant decrement is highlighted in post‐rut group for LEP gene (Figure 3B). Leptin is a hormone produced by adipose tissue functioning as an indicator of body energy reserves (Gaspar‐López et al. 2009). In male specimens, especially in seasonal animals, it connects the nutritional status to the reproductive function (Szczesna and Zieba 2015). A previous study on other cervids has already highlighted a decrease in circulating leptin levels after the rutting season, along with a substantial weight loss (Gaspar‐López et al. 2009). Our findings provide new evidence that the Roe deer follows a similar pattern. This suggests that leptin plays a consistent role in the metabolic‐reproductive axis of seasonal breeders, although the exact mechanism may vary in accordance with the species‐specific physiology. Our results on mRNA are further supported by protein concentration data, which show a similar trend in post‐rut animals (Figure 4C). Additionally, a statistically significant reduction in THBSⅡ gene expression is observed in post‐rut animals (Figure 3D), confirming preliminary data obtained on pooled samples, as shown by heatmap (Figure 1B). THBSⅡ encodes thrombospondin‐2 (TSP‐2), a well‐known inhibitor of angiogenesis (Zhang et al. 2020). While TSP‐2 has been extensively studied in the context of cancer research (Pienkowski et al. 2025), its role in spermatogenesis remains poorly understood. The observed decrease may reflect a specific molecular adaptation associated with testicular tissue remodeling following the reproductive season. However, further studies are needed to clarify the significance of these results. Notably, TSP‐2 did not show significant gene expression changes in response to photoperiod in *Peromyscus leucopus* (Pyter et al. 2005), suggesting that its regulatory role could be species‐specific. The last gene object of discussion is NRP2 that presents an opposite trend compared to the others of the same Panel (Figure 3C). Our results show a discrepancy between NRP2 mRNA and protein concentration in testis parenchyma in post‐rut, suggesting a complex regulation. NRP2 is a pleiotropic transmembrane co‐receptor, involved in binding different ligands such as 3 semaphorines (SEMA3) and VEGF playing different functions. NRP2, in fact, is considered a potential target in cardiovascular diseases (Harman et al. 2020). Additionally, it guides migration of GnRH neurons, critical for puberty and fertility (Oleari et al. 2019). Moreover, NRP2 knockout mice show reduced GnRH neurons and smaller gonads (Men et al. 2021). The observed mRNA upregulation, alongside with protein downregulation, may result from microRNA‐mediated translational repression or increased shedding of soluble NRP2 isoforms. These findings highlight previously unexplored mechanisms regulating NRP2 during seasonal reproduction and needing further investigation.

The Spearman's correlation analysis confirmed a complex interplay between testicular testosterone levels and gene expression of some of the angiogenesis and oxidative stress‐related markers analyzed. NRP2 emerges as a central node in the correlation network because of its strong positive correlations between Leptin mRNA and SOD3 mRNA. This indicates a coordinated transcriptional program potentially linking vascular remodeling with antioxidant responses. On the other hand, the negative association between leptin and SOD3 mRNA may indicate that metabolic signaling and antioxidant defense are regulated in opposite directions. A notable finding is the strong negative correlation between NRP2 mRNA and NRP2 protein, highlighting the importance of post‐transcriptional and post‐translational regulatory mechanisms. Within this gene expression network, testicular testosterone acts as a key functional modulator. Positive correlations with DHCR24 and Leptin mRNA indicate a close association between androgen production and pathways involved in lipid metabolism and cellular homeostasis. In contrast, the negative correlation between testosterone and SOD3 mRNA suggests that increased steroidogenic activity may be accompanied by reduced transcriptional antioxidant defenses, reflecting a functional shift favoring steroidogenesis and vascular support over oxidative stress protection during periods of high reproductive activity.

In such a scenario, it is interesting to note that literature specific to the analyzed genes and proteins is also lacking for other broad‐based models of seasonal reproduction, like hamsters (Phodopus sungorus and Cricetulus barabensis). Indeed, these species have been extensively used to investigate testicular involution (Munley et al. 2018; Xue, Xu, Chen, et al. 2022; Xue, Xu, Wu, et al. 2022), with several key regulatory genes been identified, including prolactin receptor (PRLR) (Xue, Xu, Wu, et al. 2022) and RFRP‐3, which acts as signal mediator between melatonin and reproductive axis activation (Xue, Xu, Chen, et al. 2022). Unfortunately, assays specifically targeting both the oxidative stress and angiogenetic pathways that would allow us to compare our results are lacking, once again proving the need for further comparative investigations.

## 5 Conclusion

This article had the objective to do an extensive and detailed analysis of two important pathways involved in the peculiar seasonal transformation that occurs in Roe deer bucks each year, namely Angiogenesis and Oxidative stress. The decision to first perform a wider analysis and then to select the most promising candidates for more specific individual analyses allowed for a focused yet comprehensive approach that highlights novel genes that have yet to be investigated. Generally speaking, it is proven that the increment of mRNA of some relevant antioxidant genes probably plays a recovery function after the reproductive intense effort. Downregulation of a metabolic reproductive regulatory gene, such as leptin, in the post‐rut period, instead, validated by its protein concentration, appears to follow the same trend as other seasonal animals. Finally, the upregulation and translation repression of NRP2 in post‐rut period, given to its pleiotropic functions, deserves further in‐depth investigation. Considering these preliminary yet substantial scientific findings, continuing to investigate the rapid and substantial changes occurring in this species becomes essential with the aim of advancing fertility research, both in animal models and in a translational framework.

## Author Contributions

**Ilaria Troisio:** methodology, formal analysis, writing – original draft. **Domenico Ventrella:** investigation, funding acquisition, writing – review and editing. **Bálint Lóránt Hausz:** methodology. **Mattia Cesauri:** methodology. **Niccolò Ian Vannetti:** methodology. **Maria Laura Bacci:** conceptualization, project administration, funding acquisition. **Alberto Elmi:** conceptualization, formal analysis, supervision, writing – review and editing. **Augusta Zannoni:** conceptualization, formal analysis, supervision, writing – review and editing.

## Conflicts of Interest

The authors declare no conflicts of interest.

## Supporting information


**Figure S1:** Heat maps showing the ∆Ct values (∆Ct = mean Ct of reference genes – Ct of the target gene), derived from genes with Ct < 35, for the pre‐rut and post‐rut groups in the Oxidative Stress (A) and Angiogenesis (B) arrays. Less negative ∆Ct values (represented by lighter colours) indicate higher gene expression. **Table S1:** Cycle thresholds (Ct) for the genes belonging to the oxidative stress commercial array. **Table S2:** Cycle thresholds (Ct) for the genes belonging to the angiogenesis commercial array.

## Data Availability

The data sets generated during and/or analyzed during the current study are available at doi. https://doi.org/10.6092/unibo/amsacta/8515.
