# Supplementary material for: Molecular Investigations on Angiogenesis and Oxidative Stress in Roe Deer (Capreolus capreolus) Bucks' Testes Throughout the Reproductive Cycle
Source: J Exp Zool A Ecol Integr Physiol. 2026 Jan 27;345(4):351–60. doi: 10.1002/jez.70067 (PMC13054778; doi:10.1002/jez.70067)

# SUPPLEMENTARY MATERIALS

**Molecular investigations on angiogenesis and oxidative stress in Roe deer (*Capreolus capreolus*) bucks’ testes throughout the reproductive cycle**

Ilaria Troisio. Domenico Ventrella. Bálint Lóránt Hausz. Mattia Cesauri. Niccolò Ian Vannetti. Maria Laura Bacci. Alberto Elmi. Augusta Zannoni

**Table S1** Cycle thresholds (Ct) for the genes belonging to the oxidative stress commercial array.

| **GENE** | **PRE-RUT POOL** | **POST-RUT POOL** | **GENE** | **PRE-RUT POOL** | **POST-RUT POOL** | **GENE** | **PRE-RUT POOL** | **POST-RUT POOL** |
| --- | --- | --- | --- | --- | --- | --- | --- | --- |
| **ALB** | 39.35 | N/A | **GSS** | 29.22 | 28.09 | **PRDX2** | 28.48 | 26.24 |
| **ALOX12** | 29.24 | 27.94 | **GSTP1** | 28.66 | 28.31 | **PRDX3** | 27.92 | 26.16 |
| **AOX1** | 28.53 | 26.08 | **GSTZ1** | 32.92 | 32.98 | **PRDX4** | 32.74 | 29.71 |
| **APOE** | 30.55 | 30.44 | **GTF2I** | 27.39 | 25.99 | **PRDX5** | 29.62 | 28.68 |
| **ATOX1** | 25.58 | 25.1 | **HMOX1** | 30.97 | 30.28 | **PRDX6** | 24.59 | 23.3 |
| **BNIP3** | 27.08 | 27.6 | **HSPA1A** | 35.29 | 34.48 | **PREX1** | 32.23 | 31.7 |
| **CAT** | 28.65 | 27.83 | **LOC788751** | 37.09 | 36.29 | **PRNP** | 27.69 | 26.01 |
| **CCL5** | 36.26 | 35.01 | **LPO** | 36.46 | 36.15 | **PTGS1** | N/A | 39.57 |
| **CCS** | 33.64 | 33.28 | **MB** | 34.79 | 35.12 | **PTGS2** | 36.25 | 35.94 |
| **CYBB** | 33.01 | 31.81 | **MBL2** | 33.11 | 32.86 | **PXDN** | 33.98 | 33.68 |
| **CYGB** | 30.83 | 29.91 | **MGST3** | 27.65 | 26.11 | **RNF7** | 35.07 | 34.4 |
| **DHCR24** | 27.41 | 27.99 | **MPO** | 37.17 | 36.28 | **SCARA3** | 29.49 | 26.71 |
| **DUOX1** | 28.66 | 28.63 | **MPV17** | 37.61 | N/A | **SEPP1** | 35.79 | 34.12 |
| **DUOX2** | 34.95 | 34.87 | **MSRA** | 28.37 | 28.12 | **SFTPD** | 39.39 | 37.32 |
| **DUSP1** | 28.63 | 28.48 | **MT3** | 38.63 | 36.31 | **SIRT2** | 30.3 | 29.59 |
| **EPHX2** | 28.66 | 27.32 | **NCF1** | 27.34 | 27.34 | **SOD1** | 25.68 | 23.92 |
| **FOXM1** | 26.4 | 26.13 | **NCF2** | 31.2 | 30.99 | **SOD2** | 29.63 | 28.11 |
| **FTH1** | 25.42 | 23.7 | **NOS2** | 31.63 | 31.05 | **SOD3** | 32.03 | 28.17 |
| **GCLC** | 34.52 | 33.39 | **NOX4** | 37.86 | 36.32 | **SQSTM1** | 29.8 | 28.87 |
| **GCLM** | 28.5 | 27.42 | **NOX5** | 35.46 | 35.64 | **SRXN1** | 28.4 | 27.09 |
| **GPX1** | N/A | N/A | **NOXA1** | 36.63 | 35.46 | **STK25** | 34.49 | 33.73 |
| **GPX2** | 30.87 | 30.51 | **NQO1** | 29.39 | 27.91 | **TPO** | N/A | 39.25 |
| **GPX3** | 36.48 | 34.25 | **NUDT1** | 37.47 | 36.4 | **TTN** | 31.05 | 30.49 |
| **GPX4** | 24.99 | 24.3 | **OXR1** | 26.54 | 26.07 | **TXN** | 39.89 | 39.32 |
| **GPX5** | 38.52 | 38.33 | **OXSR1** | 27.26 | 26.62 | **TXNRD1** | 28.25 | 27.21 |
| **GPX6** | 32.59 | 32.19 | **PDLIM1** | 30.65 | 29.69 | **TXNRD2** | 32.36 | 29.94 |
| **GPX7** | 31.46 | 30.84 | **PNKP** | 39.1 | 37.19 | **UCP2** | 27.04 | 26.91 |
| **GSR** | 30.53 | 29.59 | **PRDX1** | 25.91 | 24.66 | **VIMP** | 32.28 | 32.03 |

**Table S2** Cycle thresholds (Ct) for the genes belonging to the angiogenesis commercial array.

| **GENE** | **PRE-RUT POOL** | **POST-RUT POOL** | **GENE** | **PRE-RUT POOL** | **POST-RUT POOL** | **GENE** | **PRE-RUT POOL** | **POST-RUT POOL** |
| --- | --- | --- | --- | --- | --- | --- | --- | --- |
| **AKT1** | 27.24 | 26.25 | **FGF2** | 34.03 | 33.14 | **NRP2** | 31.37 | 28.28 |
| **ANG** | N/A | 37.54 | **FGFR3** | 33.02 | 31.92 | **PDGFA** | 30.6 | 28.7 |
| **ANG2** | 33.25 | 32.59 | **FIGF** | 34.83 | 32.59 | **PECAM1** | 38.23 | 36.98 |
| **ANGPT1** | 32.67 | 32.07 | **FLT1** | 38.96 | 37.72 | **PF4** | 35.2 | 34.76 |
| **ANGPT2** | 39.48 | 38 | **FN1** | 36.67 | 36.17 | **PGF** | 34.01 | 34.51 |
| **ANGPTL4** | 31.09 | 31.11 | **GRO1** | N/A | 39.44 | **PLAU** | 28.47 | 27.26 |
| **ANPEP** | 31.42 | 29.6 | **HGF** | 32.89 | 32.02 | **PLG** | N/A | 35.8 |
| **BAI1** | 33.18 | 32.47 | **HIF1A** | 28.07 | 26.83 | **PROK2** | 24.84 | 24.82 |
| **CCL11** | 39.64 | 39.86 | **HPSE** | 30.7 | 29.68 | **PTGS1** | 39.37 | 39.17 |
| **CCL2** | 31.4 | 31.68 | **ID1** | 36.21 | 35.24 | **S1PR1** | 40 | 34.88 |
| **CDH5** | 32.39 | 31.22 | **IFNB1** | N/A | 38.75 | **SERPINE1** | 33.26 | 33.44 |
| **COL18A1** | 36.1 | 35.35 | **IFNG** | 33.93 | 33.2 | **SERPINF1** | 33.69 | 34.54 |
| **COL4A3** | 27.64 | 26.3 | **IGF1** | 30.49 | 30.26 | **SPHK1** | 36.95 | 35.16 |
| **CTGF** | 27.56 | 26.3 | **IL1B** | N/A | 36.29 | **TEK** | 31.81 | 30.72 |
| **CXCL10** | 29.94 | 29.33 | **IL6** | 37.01 | 35.59 | **TGFA** | 32.65 | 32.62 |
| **CXCL5** | 32.42 | 31.9 | **ITGAV** | 28.29 | 25.95 | **TGFB1** | 30.71 | 29 |
| **CXCL8** | 35.81 | 36.67 | **ITGB3** | 32.96 | 31.61 | **TGFB2** | 30.22 | 29.05 |
| **CXCL9** | 31.55 | 31.35 | **JAG1** | 35.11 | 33.49 | **TGFBR1** | 34.06 | 33.43 |
| **EDN1** | 37.26 | 36.43 | **KDR** | N/A | N/A | **THBS1** | 33.01 | 31.96 |
| **EFNA1** | 38.28 | 37.27 | **LECT1** | 32.93 | 32.11 | **THBS2** | 30.91 | 31.73 |
| **EFNB2** | 29.03 | 26.85 | **LEP** | 32.02 | 32.77 | **TIE1** | 30.54 | 29.88 |
| **EGF** | 36.34 | 35.36 | **MDK** | 30.43 | 29.57 | **TIMP1** | 27.42 | 27.67 |
| **EPAS1** | 27.56 | 26.76 | **MMP14** | 28.77 | 26.88 | **TIMP2** | 27.72 | 26.12 |
| **EPHB4** | 32.24 | 31.29 | **MMP2** | 28.7 | 27.5 | **TIMP3** | 27.11 | 24.9 |
| **ERBB2** | 32.75 | 31.71 | **MMP9** | 29.46 | 28.87 | **TNF** | 37.67 | 37.49 |
| **F2** | 23.34 | 23.91 | **NOS3** | 28.7 | 27.77 | **VEGFA** | 30.4 | 30.18 |
| **F3** | 31.11 | 30.71 | **NOTCH4** | 31.46 | 30.7 | **VEGFB** | 30.5 | 28.93 |
| **FGF1** | 27.99 | 27.08 | **NRP1** | 28.26 | 27.13 | **VEGFC** | 30.92 | 30.03 |

**Figure S1** Heat maps showing the ∆Ct values (∆Ct = mean Ct of reference genes – Ct of the target gene), derived from genes with Ct < 35, for the pre-rut and post-rut groups in the Oxidative Stress (A) and Angiogenesis (B) arrays. Less negative ∆Ct values (represented by lighter colours) indicate higher gene expression.


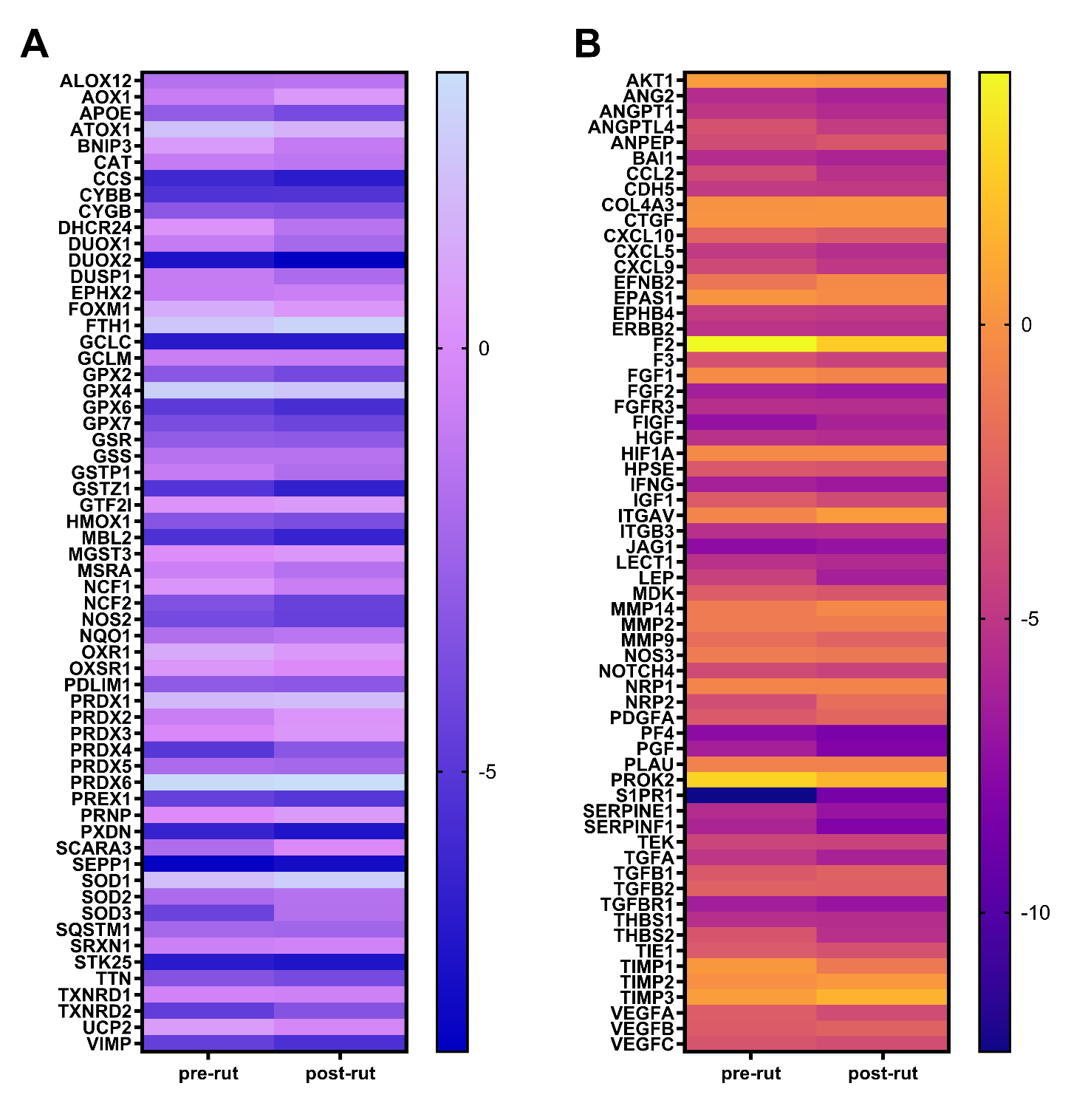

Supplement: Supplementary file 1 — Figure S1: Heat maps showing the ∆Ct values (∆Ct = mean Ct of reference genes – Ct of the target gene), derived from genes with Ct < 35, for the pre‐rut and post‐rut groups in the Oxidative Stress (A) and Angiogenesis (B) arrays. Less negative ∆Ct values (represented by lighter colours) indicate higher gene expression. Table S1: Cycle thresholds (Ct) for the genes belonging to the oxidative stress commercial array. Table S2: Cycle thresholds (Ct) for the genes belonging to the angiogenesis commercial array. [file JEZ-345-351-s001.docx]
